# Optimizing the MDS-UPDRS Part III for early-stage Parkinson’s: early supportive evidence for a limb-related bradykinesia/rigidity sub-score

**DOI:** 10.1038/s41531-025-01072-2

**Published:** 2025-10-17

**Authors:** Antoine Regnault, Maria Key Prato, Stéphane Quéré, Anne Benoit, Nathalie J. Massat, Thomas Morel

**Affiliations:** 1 Department of Statistics, Modus Outcomes, A THREAD Company, Lyon, France; 2 Department of Biometrics and Data Sciences (BDS), UCB, Brussels, Belgium; 3 Department of Statistics, UCB (contracted via Veramed Ltd, Twickenham), Slough, UK; 4 Department of Patient Centred Outcomes Research, UCB, Bulle, Switzerland

**Keywords:** Clinical trial design, Outcomes research, Neurological disorders

## Abstract

The MDS-UPDRS was not specifically developed for early-stage Parkinson’s disease (PD). This study investigated a sub-score of MDS-UPDRS Part III to assess bradykinesia and rigidity, which would be more targeted and clinically meaningful to assess disease progression in early-stage PD. A cross-sectional Rasch model was applied to the Parkinson’s Progression Markers Initiative (PPMI) untreated PD cohort data (*N* = 423) to define an adequate item set. Additionally, we evaluated how the daily life items from MDS-UPDRS Part II relate to bradykinesia and rigidity severity. A sub-score of 15 MDS-UPDRS Part III items focusing on limb-related bradykinesia and rigidity demonstrated good measurement properties in early-stage PD. Items adequately targeted to the study sample, and a meaningful hierarchy supported the validity of the scale. The scale was invariant to symptomatic treatment intake. Onset of clinical signs was represented as a milestone in the emergence of impairment in patients’ daily functioning.

## Introduction

Demonstrating efficacy of disease-modifying therapies (DMTs) in Parkinson’s disease (PD) is challenging due to the slowly progressing nature of the disease, and because of the requirement for DMT initiation as early as possible after diagnosis. To demonstrate the ability of a DMT to slow down, or even stop, the progression of clinical signs and symptoms, evidence must either be gathered over a long timeframe, which would require clinical trials spanning several years, or by using assessments responsive to very subtle changes indicative of disease progression. Due to the imperative of delivering novel therapies to people with Parkinson’s^1–3^, the option of longer trials is not an ideal solution. In contrast, improving the ability to detect very sensitive and meaningful indicators of disease progression is a critical objective for clinical research in PD.

Another challenge for the evaluation of new DMTs in PD is the impact of symptomatic treatment (ST), often initiated within the first months or years of diagnosis, which may mask signs of disease progression^4,5^. Therefore, another question of interest in relation to measures of disease progression in early-stage PD is whether, or to what extent, these measures are confounded by the initiation of ST.

The Movement Disorder Society-Unified Parkinson’s Disease Rating Scale (MDS-UPDRS) is widely used as a clinical outcome assessment to demonstrate treatment effects in clinical trials in PD^6,7^. The scale includes both patient-rated and clinician-assessed evaluations of motor aspects of the disease, in Parts II (Motor Experiences of Daily Living) and III (Motor Examination), respectively^7,8^. Originally designed as a global measure of PD severity, with all Hoehn and Yahr stages represented in its development, the MDS-UPDRS was not designed to evaluate the patient experience at any particular stage of the disease. However, with the shift in focus toward the need for intervention at earlier stages of disease, particularly in the context of DMT research, bespoke analyses have aimed to ascertain the ability of the MDS-UPDRS to assess the mildest manifestations of PD. These analyses have highlighted concerns about the ability of the MDS-UPDRS to detect disease severity in early-stage PD, as many of the items of the MDS-UPDRS Parts II and III lack relevance to this population^8–10^. This is at odds with the current ambition to evaluate meaningful aspects of concepts of interest that are relevant to daily life functioning in people with early-stage Parkinson's, as championed by regulatory bodies and Critical Path Parkinson’s^3,11–14^.

Currently, there is no consensus definition of ‘early-stage PD’ among the scientific and regulatory communities. Commonly, a working definition is generally based on time (i.e., within 3 years from diagnosis) and functional impairment (i.e., Hoehn and Yahr Stage ≤2).

An immediate actionable option to make use of the MDS-UPDRS in early-stage PD clinical trials is to identify items that are both the most sensitive to detect change and the most clinically meaningful to early-stage PD and translate them into “spin-off” measures, without amending the content, structure, or mode of administration of the MDS-UPDRS^10^. By focusing on these items, it is expected that the signal-to-noise ratio would be improved or optimized, and the resulting measure would be more sensitive to disease progression^9^. Such an approach has been recognized by regulatory agencies as a pathway to pragmatically expedite the use of fit-for-purpose clinical outcome assessments in early-stage PD for current drug development^10,15^.

Bradykinesia, rigidity, and rest tremor are three key features of PD that have been repeatedly identified as central to the patient experience of early-stage PD in qualitative studies^16–23^. Worsening of these motor signs may thus form a natural candidate to detect progression in early-stage PD. The MDS-UPDRS Part III includes several items assessing investigator-rated severity of these motor signs^7^. It may therefore be possible to develop a measure from these MDS-UPDRS items that would be better suited for use in the context of early-stage PD, compared with the MDS-UPDRS items that are currently used.

Our primary objective was to explore the possibility of generating outcome measures focused on early motor signs of PD, using only those MDS-UPDRS Part III items identified as characteristic of early-stage PD and specifically focusing on those signs visible in the limbs. As asymmetry of motor signs is a cardinal feature of PD diagnosis, which impacts phenotypic expression and progression^24,25^ and bilaterality is a key clinical staging milestone in early PD progression^26^, we hypothesized that assessing motor signs that can be observed on both sides of the body may improve the ability to detect clinically meaningful change. Additional objectives were to explore the invariance of such measures to ST intake, and to understand how the severity of the motor signs and symptoms in early-stage PD (as measured by MDS-UPDRS Part III) relate to the patient-reported impacts measured by MDS-UPDRS Part II.

To address these objectives, we applied robust psychometric methods to the well-characterized cohort of early untreated PD participant's from the open-access Parkinson’s Progression Markers Initiative (PPMI) 001 database^27,28^.

## Results

### Study sample

In the cohort of 423 participants with untreated PD, approximately two-thirds of participants were male and mostly white (92%)^28^. The mean (± standard deviation [SD]) age was 62 ± 10 years (range: 34–85; Table 1). Median time from PD diagnosis was 4.0 months (interquartile range 2.0–8.0). At diagnosis, 82% of participants had bradykinesia (Table 1). The majority (89%) of participants were right-handed, while the side affected at PD onset was almost evenly balanced (55% on the right vs. 42% on the left side). The sample was evenly distributed between the Hoehn and Yahr Stages 1 and 2 (Table 1). At baseline, all participants were ST-naïve; details on the type of levodopa or dopamine agonist used have been reported previously^28^. Participant demographics, including sex, ethnicity, and family history, have also been described in previous publications^28,29^.

**Table 1:** Sample demographics and clinical characteristics on enrollment to PPMI

	Participants with untreated PD (*N* = 423)
Sex, *n* (%) Male Female	277 (65.5) 146 (34.5)
Age, years Mean (SD) Median (IQR) Range	62.11 (9.70) 63.00 (55.25–69.33) 34.16–85.25
Time from PD diagnosis, months Mean (SD) Median (IQR) Range	6.56 (6.49) 4.01 (2.04–7.98) 0–35.98
Bradykinesia at diagnosis, *n* (%) Yes No Missing	348 (82.3) 72 (17.0) 3 (0.7)
Handedness, *n* (%) Right Left Mixed	375 (88.7) 38 (9.0) 10 (2.4)
Correspondence between side predominantly affected at onset and handedness, *n* (%) Same side (ipsilateral) Other side (contralateral) Bilateral	235 (55.5) 169 (40.0) 19 (4.5)
Hoehn and Yahr stage, *n* (%) 1 2	207 (48.9) 216 (51.1)

*IQR* interquartile range, *PD* Parkinson’s disease, *PPMI* Parkinson’s Progression Markers Initiative, *SD* standard deviation.

### Description of MDS-UPDRS Part III responses

**Fig. 1:**
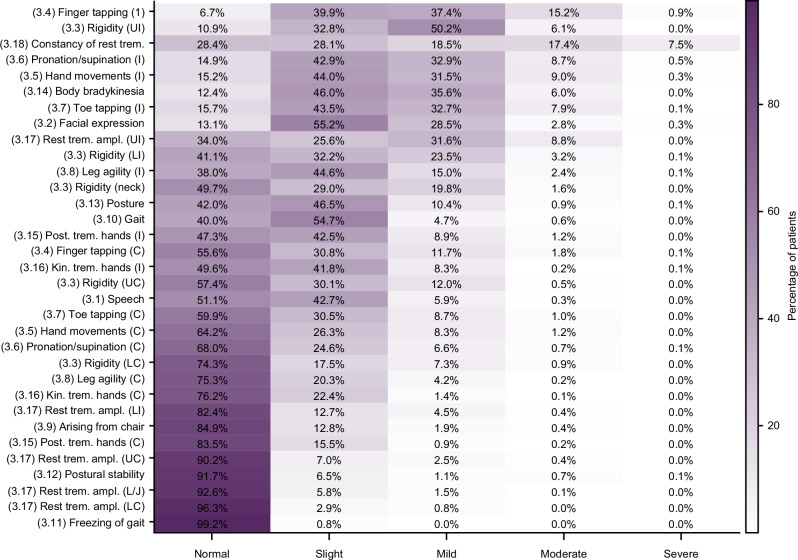
Distribution of MDS-UPDRS Part III item responses in participants with untreated (ST-naïve) PD (pooled data from assessments performed at screening, baseline, month 3, month 6, month 9, month 12, month 18, and month 24; *N* = 1906). Each cell of the heatmap shows the percentage of participants rated at the given severity (column) for the given item (row). Darker fill colors indicate higher percentages. *C* contralateral, *I* ipsilateral, *Kin. trem*. kinetic tremor, *LC* contralateral lower limb, *LI* ipsilateral lower limb, *L/J* lip/jaw, *MDS-UPDRS* Movement Disorder Society-Unified Parkinson’s Disease Rating Scale, *PD* Parkinson’s disease, *Post. trem*. postural tremor, *Rest trem. ampl.* rest tremor amplitude, *ST* symptomatic treatment, *UC* contralateral upper limb, *UI* ipsilateral upper limb.

A total of 2962 assessments were performed for the 423 participants in this cohort between screening and month 24, of which 1906 assessments were completed prior to initiation of ST. The distribution of responses was weighted towards mild symptoms (Fig. 1), with very few participants exhibiting symptoms rated as severe on any item. For this reason and for the subsequent needs of the Rasch measurement theory (RMT) analyses, ‘moderate’ and ‘severe’ responses were collapsed into a single category (‘moderate/severe’), as discrimination between the two categories in the Rasch model would not be possible with so few participants assigned to each; response options were therefore rescored on a 4-point scale.

### MDS-UPDRS-derived measure of severity of motor signs specific to early-stage PD: limb bradykinesia and rigidity, and rest tremor

The set included items related to limb bradykinesia, rigidity, and rest tremor (i.e., 21 items out of 33 items in the full MDS-UPDRS Part III) and showed an adequate targeting of the scale to the sample (Supplementary Fig. 1a), although a few item thresholds were distributed towards the more severe end of the continuum, where no individual observation was present (i.e., no observation in the sample corresponded to such high severity of symptoms). A gap was observed in the coverage of the milder motor signs. Reliability was adequate, with an estimated reliability coefficient (PSI) of 0.80.

Limb bradykinesia and rigidity items did not show disordered thresholds, and the majority of items were within the recommended range for fit residuals. However, all rest tremor items showed disordered thresholds (Table 2), suggesting that the response scale for these items did not work as intended. Furthermore, all rest tremor items except one had both a standardized fit residual statistic outside the –2.5 to +2.5 recommended range and a statistically significant chi-squared value (Table 2), indicating deviation from the Rasch model. Therefore, rest tremor did not fit well on a single severity continuum with bradykinesia and rigidity, suggesting that these two groups of motor signs (bradykinesia/rigidity and rest tremor) should be explored independently in separate RMT analyses.

**Table 2:**
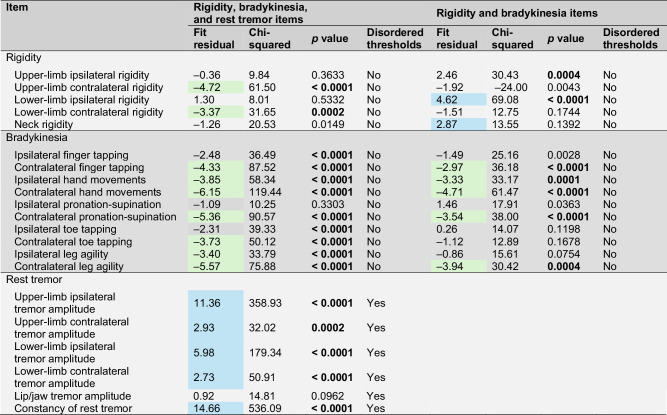
Item performance for MDS-UPDRS-derived measures of severity of motor signs in RMT analyses of selected items relevant to early-stage PD

Fit residuals recommended range is –2.5 to +2.5; values highlighted in blue are >+2.5, indicating items under-discriminating the measured concept; values highlighted in green are <–2.5, indicating items over-discriminating the measured concept.

Chi-square probabilities were reported before Bonferroni adjustment; those significant after Bonferroni adjustment at *p* < 0.01 are shown in bold.

*N* = 1906 MDS-UPDRS assessments in 423 participants.

*MDS-UPDRS* Movement Disorder Society-Unified Parkinson’s Disease Rating Scale, *PD* Parkinson’s disease, *RMT* Rasch measurement theory.

### MDS-UPDRS-derived measure of severity of limb bradykinesia and rigidity

**Fig. 2:**
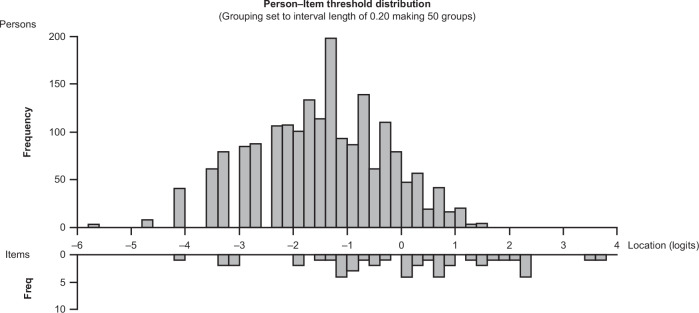
Scale-to-sample targeting of the MDS-UPDRS Part III bradykinesia and rigidity item set in participants with untreated (ST-naïve) PD (pooled data from assessments performed at screening, baseline, month 3, month 6, month 9, month 12, month 18, and month 24; *N* = 1906). The upper panel shows the distribution of the individual observations over the continuum of bradykinesia/rigidity severity; the lower panel shows the distribution of the item thresholds, i.e., the boundaries between adjacent item response categories, on the continuum of bradykinesia/rigidity severity. *Freq* frequency, *MDS-UPDRS* Movement Disorder Society-Unified Parkinson’s Disease Rating Scale, *PD* Parkinson’s disease, *ST* symptomatic treatment.

The Rasch model, including items relating to limb bradykinesia and rigidity only (i.e., 15 items), showed good performance with adequate scale-to-sample targeting (i.e., no floor or ceiling effect), although a gap in the coverage of the milder motor signs persisted (Fig. 2; Supplementary Fig. 1b). Reliability was also adequate, with a PSI of 0.84. No items had disordered thresholds, confirming that the response scales of these items were working as intended (Table 2). Some items had statistics suggesting possible deviation from the Rasch model (significant chi-squared values [*p* < 0.01]; Table 2), but visual inspection of the item characteristic curves (ICCs) suggested that the deviation was not strong enough to cause major limitations to the validity of considering these items as a single scale.

**Fig. 3:**
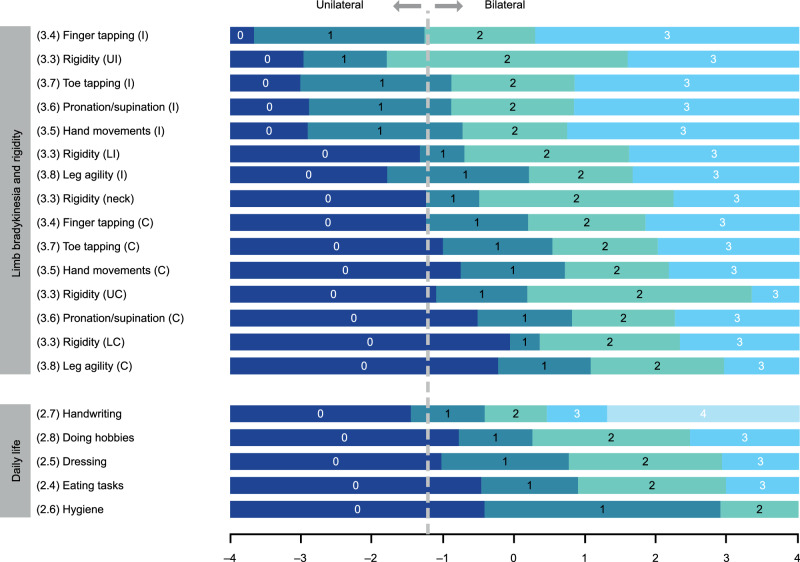
Hierarchy of MDS-UPDRS Part III limb-related bradykinesia and rigidity items from the Rasch model, with mapped MDS-UPDRS Part II daily life items. The hierarchy of Part III items (upper panel) shows ipsilateral symptoms ahead of contralateral symptoms on the severity continuum. The dotted line shows onset of bilaterality (contralateral involvement), which coincides with an impact on daily life activities starting to be reported (transition from 0 to 1 for Part II item responses; lower panel). Original response options for all Parts II and III items are 0 = ‘Normal’, 1 = ‘Slight’, 2 = ‘Mild’, 3 = ‘Moderate’, and 4 = ‘Severe’. For analysis purposes, some categories were collapsed: ‘Moderate’ and ‘Severe’ for Part II items ‘Eating tasks’ and ‘Dressing’ and all Part III items (recoded 3); ‘Mild’, ‘Moderate’, and ‘Severe’ for Part II item ‘Hygiene’ (recoded 2), and ‘Mild’ and ‘Moderate’ for Part II item ‘Doing hobbies’ (recoded 2 and ‘Severe’ recoded 3). *C* contralateral, *I* ipsilateral, *LC* contralateral lower limb, *LI* ipsilateral lower limb, *MDS-UPDRS* Movement Disorder Society-Unified Parkinson’s Disease Rating Scale, *UC* contralateral upper limb, *UI* ipsilateral upper limb.

When ordered based on the Rasch model item parameter estimates, items defined a meaningful severity continuum, providing supportive evidence for the validity of the scale: signs of bradykinesia and rigidity showed first in the ipsilateral upper limb, second in the ipsilateral lower limb, and finally in the contralateral limbs (Fig. 3, upper panel).

### MDS-UPDRS-derived measure of severity of rest tremor

In the model that included items relating to rest tremor only (i.e., six items), more than 25% of the assessments showed no evidence of rest tremor, and there were major gaps in the coverage of the rest tremor severity continuum, indicating strong mistargeting of the items to the sample (Supplementary Fig. 1c). Reliability was unsatisfactory, with an estimated reliability coefficient of 0.36. Four out of six items still had disordered thresholds, suggesting that the response scale was not working as intended. The item ‘Constancy of rest tremor’ had both standardized fit residual statistics outside the –2.5 to +2.5 recommended range and a statistically significant chi-squared value (Supplementary Table 1), indicating deviation from the Rasch model.

As the MDS-UPDRS-derived measure of severity of rest tremor did not demonstrate good properties, subsequent analyses were only conducted with the MDS-UPDRS-derived measure of severity of limb-related bradykinesia and rigidity.

### Invariance of measurement of MDS-UPDRS-derived measure of limb bradykinesia and rigidity sub-score to symptomatic treatment intake

The Rasch model was applied to the limb-related bradykinesia and rigidity items using data from a total of 3219 assessments performed within 4 years of participants' diagnoses, including 1947 assessments performed before ST intake, 874 assessments performed in the ON state after intake of levodopa (+/– other ST; within 2 h after last dose), and 398 assessments performed in the OFF state after intake of levodopa (+/– other ST; at least 6 h since last dose). The results of the RMT analysis were consistent with those observed using only assessments collected before ST initiation—coverage of the continuum by the bradykinesia and rigidity items was similar, with similar meaningful hierarchy among items along the severity continuum and no items displaying disordered thresholds. The model also showed good reliability (estimated reliability coefficient 0.84). Some items had fit statistics suggesting possible deviation from the Rasch model (slight under-discrimination of ipsilateral rigidity and over discrimination of measures of bradykinesia, particularly on the contralateral side), but ICCs suggested that the deviation was not strong enough to cause major limitations to the validity of considering these items as a single scale.

**Fig. 4:**
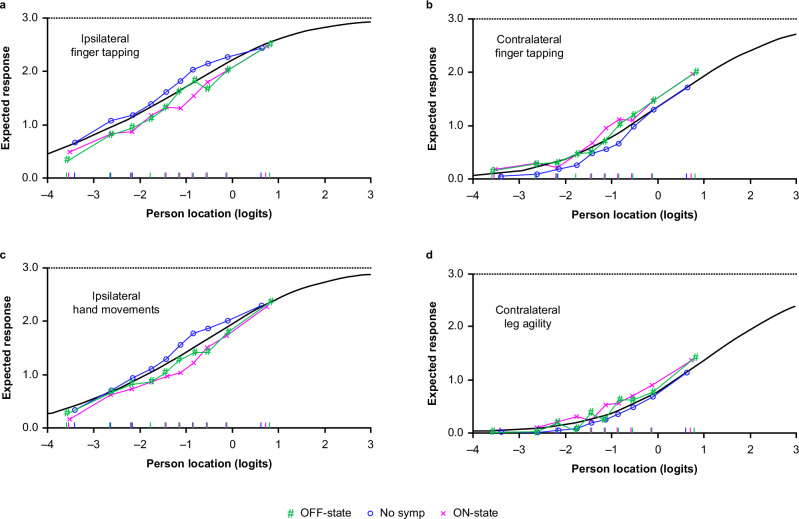
DIF of items showing high F-statistics—expected response to the items as a function of the overall bradykinesia/rigidity severity according to ST intake (no ST, ON state, or OFF state). Items: ipsilateral finger tapping (**a**), contralateral finger tapping (**b**), ipsilateral hand movements (**c**), and contralateral leg agility (**d**). The *X*-axis represents the measurement continuum of the trait (i.e., construct under measurement), with decreasing levels from left to right. The gray line represents the predicted response to the item by the Rasch model. The colored lines show the observed mean responses to the item in “class intervals” for each subgroup of the factor variable (here ST intake status). *DIF* differential item functioning, *ST* symptomatic treatment, *symp* symptom.

A few items were identified as having possible uniform differential item functioning (DIF) according to ST status, suggesting that responses to these items may be different depending on whether the respondent has initiated ST or not, and whether the measurement was taken in the ON state or OFF state after levodopa intake. These items included ipsilateral finger tapping, ipsilateral hand movements, contralateral finger tapping, and contralateral leg agility. ICCs of items with possible DIF were investigated further (Fig. 4). A marginal difference in the average response between assessments made prior to or after initiation of ST was observed: at a given level of severity of bradykinesia and rigidity, participants who were not taking ST were slightly more likely to have higher ratings for ipsilateral finger tapping and hand movements than participants on ST, while ratings for contralateral finger tapping and leg agility were slightly higher for participants on ST (with assessment taken in the ON state) than for participants assessed prior to initiation of ST.

**Fig. 5:**
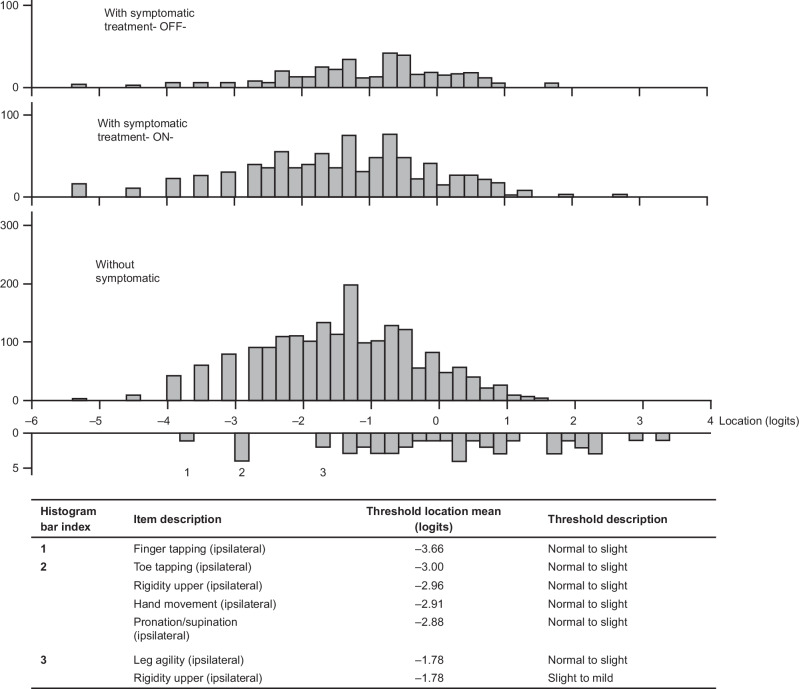
Distribution of the assessments, according to ST intake, on the severity continuum defined by the Rasch model for the MDS-UPDRS Part III limb-related bradykinesia and rigidity item set. The item thresholds impacted earliest on the continuum of PD severity are detailed out in the table below. *MDS-UPDRS* Movement Disorder Society-Unified Parkinson’s Disease Rating Scale, *PD* Parkinson’s disease, *ST* symptomatic treatment.

The distribution of assessments on the continuum of severity defined by the Rasch model for the MDS-UPDRS Part III bradykinesia and rigidity item set was plotted according to ST intake (no ST, OFF state, and ON state; Fig. 5). ST status was not associated with a shift in overall severity, but assessments taken after initiation of ST showed slightly higher variability, with mean (SD) bradykinesia/rigidity severity location of –1.405 (1.14) prior to ST initiation, and –0.950 (1.32) and –1.356 (1.40) after ST initiation assessed in the OFF, and ON states, respectively.

Overall, the expected rating to each item of the scale for participants receiving ST, in either the OFF or ON state, was not different from the expected rating for a participant with the same overall level of severity who was not receiving ST. Thus, the measure of bradykinesia and rigidity severity can be considered invariant to ST intake based on the results of this analysis.

### Mapping the MDS-UPDRS Part II daily life items on to the MDS-UPDRS Part III limb bradykinesia and rigidity continuum

The Rasch model was applied to the MDS-UPDRS Part II daily life items “anchored” on the MDS-UPDRS Part III bradykinesia/rigidity continuum. Estimated thresholds for ‘Daily life’ items from the MDS-UPDRS Part II are shown in Fig. 3, alongside estimated thresholds for severity of bradykinesia and rigidity items to illustrate the mapping of impact on daily life onto bradykinesia and rigidity severity metrics. Mapping showed that impact on daily life may start being reported when bradykinesia and rigidity are documented for the contralateral side of the body (indicated by a dotted line on Fig. 3), i.e., when early-stage PD manifestations become bilateral. This finding suggests that onset of bilaterality, in addition to being a well-documented clinical staging milestone of early PD progression^26^, also represents a tipping point with regard to impairment in daily functioning.

‘Handwriting’ was the only daily life item for which ‘slight’ issues were typically reported prior to onset of bilaterality, which is unsurprising given that correspondence between handedness and side affected at PD onset is more relevant to writing ability than bilaterality. The accumulation of signs affecting the ipsilateral upper limb (finger tapping, rigidity, pronation/supination, and hand movements) appears to be necessary before the impact on handwriting is reported.

**Fig. 6:**
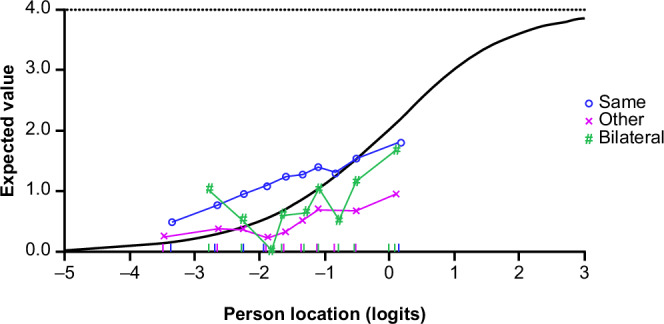
Differential Item Functioning for ‘Handwriting’ item—expected response to ‘Handwriting’ item of the MDS-UPDRS Part II as a function of the overall bradykinesia/rigidity severity according to correspondence between predominant side of PD and participant handedness. The *X*-axis represents the measurement continuum of bradykinesia and rigidity severity, as measured by the bradykinesia and rigidity item set from the MDS-UPDRS Part III. The gray line represents the predicted response to the ‘Handwriting’ item by the Rasch model. The colored lines show the observed mean responses to the ‘Handwriting’ item in ‘class intervals’ for each subgroup of the factor variable (here correspondence between handedness and predominant side of PD). *MDS-UPDRS* Movement Disorder Society-Unified Parkinson’s Disease Rating Scale, *PD* Parkinson’s disease.

Of note, the fit of some of the MDS-UPDRS Part II items to the Rasch model of bradykinesia/rigidity severity was not optimal; in particular, ‘Handwriting’ and ‘Doing hobbies’ showed under-discrimination (fit residuals >+2.5; chi-squared *p* < 0.0001). ICCs confirmed under-discrimination for the ‘Handwriting’ item: the observed response was higher (more severe) than expected in participants with low levels of bradykinesia and rigidity, and lower (less severe) than expected for those with higher severity of bradykinesia and rigidity. In addition, the ‘Handwriting’ item showed DIF for the correspondence between participant handedness and side predominantly affected at PD onset (Fig. 6): at a given level of bradykinesia and rigidity, participants for whom the side that was predominantly affected at PD onset was the same as their dominant hand were consistently more likely to report higher impact on handwriting than participants whose non-dominant side was initially affected.

## Discussion

Our analyses of a large data set of MDS-UPDRS observations from the PPMI study support the possible use of the subset of 15 limb-related bradykinesia and rigidity items from the MDS-UPDRS Part III to calculate a score for severity of bradykinesia and rigidity, which would be more targeted and clinically meaningful to early-stage PD than the total MDS-UPDRS Part III score. Our findings also suggest that the quality of measurement of bradykinesia and rigidity severity by this item set is not strongly disrupted by the initiation of ST, in particular levodopa, which is now recommended in the management of early-stage PD motor symptoms^4,30^. Finally, we have also shown that bradykinesia and rigidity typically only start impacting a patient’s daily functioning when PD becomes bilateral (i.e., when the bradykinesia and rigidity affect both sides of the body).

This research builds on a previous RMT analysis of the full 33-item MDS-UPDRS Part III in an early-stage PD population, which showed that the full item set broadly works together, but identified limitations including floor effects and a gap in coverage at the mildest end of the severity continuum for motor symptoms^9^. The results presented here are consistent with this previous research and extend it by exploring selected items of greatest relevance to early-stage PD (bradykinesia, rigidity, and rest tremor). They revealed a split between bradykinesia/rigidity symptoms and tremor symptoms, which was shown by recently published research that also documented separation between tremor and non-tremor MDS-UPDRS Part III items^31,32^. These findings consolidate the concept of creating a specific limb-related bradykinesia and rigidity score intended for use in the context of early-stage PD^33^. Additionally, the validity of this sub-score was strengthened by the RMT analysis, which revealed that the bradykinesia and rigidity item hierarchy aligned with the clinical description of PD progression. Specifically, the items corresponding to the upper limbs are the first affected, followed by those corresponding to the lower limbs^34–36^. According to the hierarchy observed in our results, the signs of rigidity appear concomitantly to those of bradykinesia and align well with the other markers of PD progression (i.e., initially manifesting in upper limbs, followed by the lower limbs, and subsequently being displayed bilaterally). This suggests that evaluating both rigidity and bradykinesia together during the early stages of PD is a reasonable approach and would facilitate a more accurate detection of early PD progression. This bradykinesia and rigidity sub-score could also be seen as an extension of the earlier UPDRS bradykinesia sub-scale, which consisted of only five bradykinesia items from the motor examination of the original UPDRS version^37^.

A gap, however, persists at the mild end of the severity continuum for bradykinesia and rigidity, as items relating to the mildest symptoms of early-stage PD are lacking. More granular assessments of upper-limb bradykinesia and rigidity, for example, by unpacking the various components of the MDS-UPDRS items assessing upper-limb bradykinesia and rigidity (which all assess several independent features under the umbrella of a single item) or the addition of new items, may allow for the creation of a more sensitive measure. Using patient-reported outcome (PRO) items could be another option to improve the detection of meaningful early signs of disease progression in early-stage PD; this solution would be fully aligned with the increasingly recognized value of PROs for the evaluation of treatment benefit in clinical trials, notably by regulatory bodies^11–13^. However, the patient-reported items comprising the MDS-UPDRS Part II do not appear to provide better detection of very mild motor manifestations^9^, so they are likely not sufficient to bridge the identified gap for measurement in early-stage PD.

Indeed, our mapping analysis showed that impacts on Part II domains relating to daily life only became apparent (slight impairment reported by participants) at the point when limb bradykinesia and rigidity signs began to manifest in the contralateral side (i.e., when PD becomes bilateral). This suggests that, in addition of being a clinical staging milestone of PD progression (as found in the Hoehn and Yahr rating system, for instance), we showed that the onset of bilaterality also represents a milestone for people with early-stage Parkinson's in the emergence of impairment in daily functioning, as measured by the MDS-UDPRS Part II. The exception to this observation was handwriting, for which impact was, unsurprisingly, related largely to the correspondence between handedness and side affected by PD symptoms. The importance of the dominant or non-dominant side was also noted in recent research^33^.

Targeted and novel PRO measures assessing meaningful changes in daily functioning in early-stage PD could be added to complement existing MDS-UPDRS items to define an improved and meaningful continuum of disease severity in the first few years post diagnosis^33^: targeted PRO measures may allow capturing of subtle changes that emerge in the patient day-to-day experience and that may not be reflected in clinical evaluation, hence providing unique information for the evaluation of disease progression in early-stage PD.

Finally, our findings indicate that our specific limb-related bradykinesia and rigidity score measures the same concept, and in the same way, before and after initiation of ST (“measurement invariance”). The wide range of levodopa formulation and dosage used across the >50 clinical sites that participated in the PPMI study reinforces our findings regarding the invariance of the score measurement to ST intake. This suggests that measurement of limb-related bradykinesia and rigidity in early-stage PD populations could, in principle, be performed using this score, whether patients have initiated ST or not, and whether they are measured in the ON or OFF state, with the results that can be compared. This is an important property to warrant the use of the score in a setting where ST could be initiated, like clinical trials in early-stage PD.

Limitations of the analyses reported herein include reliance on a single sample. The PPMI cohort of early untreated PD is a valuable source of observational data, which has already been used to gain better understanding of the natural history of PD progression. The imbalance of males to females in the cohort is reflective of the sex differences in PD prevalence reported in North America and Europe^38^; however, the cohort is limited in its ethnic diversity^39^, and verification in an additional independent sample, ideally comprising people with early-stage Parkinson's participating in a clinical study, is currently lacking. Confirmatory research is currently ongoing, using data from clinical trials that recruited participants with early-stage PD. Additionally, our proposed sub-score does not capture rest tremor, even though it is one of the cardinal symptoms of early-stage PD^19,22,32^ and a diagnostic feature of PD; items measuring tremor amplitude and constancy were excluded because they were detrimental to its performance as a good measure with meaningful hierarchy of items. This raises the question of the holistic consideration of motor signs in the evaluation of disease progression in early-stage PD, and specifically how bradykinesia/rigidity signals can be combined with those from rest tremor. Compared with other motor symptoms, tremor displays distinctive prevalence patterns and pathobiology from other motor symptoms^40–42^, and was shown to respond differently to treatment in early-stage PD^43^. At the population level, signs of tremor are only present in a subset of people who are newly diagnosed with Parkinson's, with up to 30% of those diagnosed with early-stage disease not experiencing any sign of rest tremor. A recent study assessed the measurement performance of the MDS-UPDRS tremor items and concluded that the combination of these items did not provide a cohesive sub-score capable of capturing changes in tremor in early-stage PD^44^. These findings emphasize the need for further research to determine the most effective methods for measuring the presence and evolution of tremor in early-stage PD, and how best to combine these methods with a measure of bradykinesia and rigidity to optimize detection of progression in early-stage PD.

The promising psychometric profile of the proposed sub-score composed of 15 MDS-UPDRS Part III items focused on limb bradykinesia and rigidity may translate into improved sensitivity to detect disease progression, which is a critical factor to be considered when determining suitable endpoints for treatment evaluation in clinical trials aiming to delay or slow progression^45,46^. Among the full MDS-UPDRS, items specific to motor sign severity (i.e., MDS-UPDRS Part III items) have been shown to be the most responsive to changes related to progression in early-stage PD^47^. In this context, using a measure based on the motor signs that are most specific to early-stage PD may be a better method for capturing the effect of treatment in these early stages. Ongoing randomized clinical trials in early-stage PD populations and their open-label extension studies will offer valuable information in this regard; however, it remains of critical importance to demonstrate that a change in bradykinesia and rigidity is clinically meaningful. To address this evidence gap, we are currently conducting further research through both quantitative analysis and, in collaboration with individuals with early-stage PD as well as movement disorder specialists, a qualitative review of the clinical meaningfulness of all MDS-UPDRS Parts II and III items. Nonetheless, the slow progression of PD and its associated observable manifestations make this demonstration challenging. Investigating the MDS-UPDRS scores, particularly the bradykinesia and rigidity sub-score, over a longer timeframe, will help document how changes in the sub-score are associated with a meaningful change in the patient experience. This approach may also reveal how the sub-score connects to measures of motor signs that are more relevant in the later stages of PD. Finally, it has been demonstrated that inter-rater variability can limit the utility of clinician-rated scales such as the MDS-UPDRS as research tools^26,48,49^. The proposed sub-score could also suffer the same limitations, and consequently, to optimize inter-rater reliability, it will be essential to provide clinicians with the necessary training.

In conclusion, a score calculated with the 15 MDS-UPDRS Part III items focusing on the severity of limb-related bradykinesia and rigidity motor signs may be a good candidate for use to support the definition of endpoints for clinical trials of DMTs in PD. This new bradykinesia/rigidity sub-score showed good measurement properties in a sample of participants with untreated PD and stability to initiation of ST, suggesting that it could capture change in the context of trials targeting disease progression in early-stage PD. Yet, this sub-score may not be considered on its own in the context of demonstrating the effects of DMTs on the sub-score, as some limitations remain regarding the coverage of the mildest severity of manifestations of PD. In addition, the sub-scale does not capture all of the important motor signs in early-stage PD, such as rest tremors. Further research on innovative measurement solutions is warranted to address these limitations.

## Methods

### Study sample: the PPMI untreated PD cohort

The PPMI is a collaboration between the Michael J. Fox Foundation and researchers, clinicians, funders, and study participants working toward the goal of identifying progression biomarkers to accelerate therapeutic trials (https://www.ppmi-info.org)^50^. PPMI Study 001^51^ is a longitudinal, observational, multicenter natural history study following several cohorts. Our study utilized the PPMI untreated PD cohort, which includes 423 participants with untreated PD (recently-diagnosed with sporadic PD with a positive dopamine transporter single-photon emission computed tomography [SPECT] scan, who had not begun treatment at the time of enrollment, and without pathogenic genetic variant[s] in *LRRK2*, *GBA,* or *SNCA*) recruited from over 50 clinical sites primarily located in North America and Europe. Full inclusion criteria for this cohort have been published previously^51,52^, and include asymmetric resting tremor or asymmetric bradykinesia, or two of bradykinesia, resting tremor, and rigidity, with diagnosis within 2 years.

### Ethics and patient consent

The PPMI study was conducted in accordance with the Good Clinical Practice (GCP) and the International Conference on Harmonization (ICH) guidelines and applicable national and local regulations. All participants provided written informed consent for the procedures and requirements of the study, together with any potential hazards/risks read by and/or explained to each subject. The participants were assured of the freedom to withdraw from the study at any time.

### Analyses

Three sets of analyses were carried out using data from MDS-UPDRS assessments conducted in participants in the untreated PD PPMI cohort to address our research objectives: identify the items of the MDS-UPDRS Part III that may enable creation of adequate measures of the severity of motor signs specific to early-stage PD; investigate the invariance in measurement of the studied measures of severity of motor signs specific to early-stage PD depending on ST intake; and explore the association between the severity of motor signs specific to early-stage PD and patient perceived experience (as evaluated by MDS-UPDRS Part II items). These analyses are described below. Analyses were carried out using RUMM 2030 software (RUMM Laboratory Pty Ltd., Perth, Australia). To produce the new sub-score, data from the standard assessment of MDS-UPDRS was used without modification of the instructions or rating protocol.

### Selection of MDS-UPDRS Part III items targeted to early-stage PD

The first analysis used MDS-UPDRS assessments made at screening, baseline, and months 3, 6, 9, 12, 18, and 24 excluding any assessments performed after initiation of ST with levodopa or dopamine agonists (*N* = 1906 assessments for 423 participants included in the analysis). The motor signs expected to be relevant to early-stage PD were selected for inclusion in the analysis based on three elements: (i) their relevance to the patient experience of early-stage PD, after clinical and conceptual review of the MDS-UPDRS Part III items (17, 18), (ii) their ability to be measured on either side of the body to capture laterality of disease manifestations^26,53^, and (iii) according to the item clusters established through factor analyses in the original MDS-UPDRS clinimetric evaluation (keeping only the factors related to ‘rest tremor’ [factor 2], ‘rigidity’ [factor 3], ‘bradykinesia right upper extremity’ [factor 4], ‘bradykinesia left upper extremity’ [factor 5], and ‘lower-limb bradykinesia’ [factor 7])^7^. As a result, MDS-UPDRS Part III items corresponding to limb bradykinesia, rigidity, and rest tremor were selected and analyzed in the RMT framework^54,55^ to evaluate if they could form a meaningful measure of motor signs targeted for early-stage PD. MDS-UPDRS Part III items measured in the right and left limb were processed before inclusion in the model to reflect the rating of the ipsilateral side (i.e., the side of the body predominantly affected by PD at symptom onset) or the contralateral side (i.e., the side of the body not predominantly affected by PD at symptom onset).

### Statistical analyses

An iterative process was conducted using the Rasch model with this initial selection of items to identify an item set that was conceptually cohesive (i.e., including items reflecting a clearly delineated group of symptoms) and that had satisfactory measurement properties according to the following properties documented by the Rasch model:

- Targeting: scale-to-sample targeting, which concerns the match between the range of the target concept measured by the item set and the range of the target concept in the sample of participants. This is assessed by examining the spread of person and item locations in these two relative distributions. This analysis informed how suitable the sample is for evaluating the item set and how suitable the item set is for measuring the sample characteristics^56^.
- Fit: items must work together (fit) to define a clinically and statistically meaningful score. Otherwise, it is inappropriate to sum item responses to reach a total score and consider the total score an accurate measure of each target concept. When items do not work together in this way (i.e., there is item misfit), the validity of an item set is questionable. Evidence for item fit is based on natural ordering of item response options (ordering of item thresholds)^57^, statistical indicators (standardized fit residual, chi-squared), and graphical indicators (ICC)^56^. As a rule of thumb, standardized fit residual values are recommended to lie in the range –2.5 to +2.5^58^.
- Dependency: the response to any item in the item set should not directly influence the response to any other in the item set^59^. If this happens, measurement estimates may be biased, and reliability may be artificially elevated. RMT determines this effect by examining residual correlations.
- Reliability: reliability is assessed using the PSI^60^, a reliability coefficient estimate. Reliability coefficients >0.8 are deemed acceptable^55^.
- Measurement invariance: this is assessed by searching for the presence of DIF between predefined subgroups of interest. DIF is detected if the expected response to the item under consideration differs for two individuals who share a common level on the measured concept but belong to different groups (e.g., male vs. female). DIF is investigated using a two-way analysis of variance (ANOVA) of residuals, with the group of interest for the DIF analysis and “class interval” as explanatory variables^61^. Class interval is an ordinal variable, which splits the sample into groups of equal size according to the estimated person locations. DIF is suspected if the residual mean differs significantly between DIF groups (uniform DIF). If the interaction term between the DIF group and the class interval is also significant, non-uniform DIF is suspected. Visual inspection of the mean responses observed in the DIF groups across class intervals provides interpretation guidance on the direction of possible DIF.

### Invariance of measurement depending on ST intake

Analyses were performed to explore the invariance to ST intake of the MDS-UPDRS-derived measure of severity for items identified in the first analysis. The selected MDS-UPDRS Part III items were included in the Rasch model, using all assessments recorded within 4 years of a participant's diagnosis (*N* = 3219), expanding the analysis timeframe to incorporate assessments performed before and after initiation of ST. DIF was evaluated according to ST status at the time of the assessment: no ST (assessments performed prior to initiation of ST); ST “OFF state” (assessments performed >6 h since last dose of dopaminergic therapy, in participants who have commenced ST); or ST “ON state” (assessments performed 1–2 h after receiving medication in clinic).

### Mapping the patient experience onto the continuum of motor signs severity specific to early-stage PD

An ‘anchored’ Rasch model was used to explore the association between severity of limb-related motor signs specific to early-stage PD (i.e., MDS-UPDRS Part III items identified in the iterative analysis described above) and impact of motor signs on participants' daily lives, as measured by MDS-UPDRS Part II domains specifically related to daily activities (i.e., ‘Handwriting’, ‘Eating tasks’, ‘Dressing’, ‘Hygiene’, and ‘Doing hobbies’). The parameters for the MDS-UPDRS Part III items included in the model were fixed at the values estimated in the first analysis. The daily life items from the MDS-UPDRS Part II were rescored prior to inclusion in the model^9^; indeed a previous RMT analysis of MDS-UPDRS Part II items demonstrated that several items had disordered thresholds, indicating the presence of response options that participants could not distinguish properly. Therefore, response options that conceptually fit together were merged, and items were rescored on the resulting collapsed scales: ‘Eating tasks’ and ‘Dressing’ items were rescored on a 4-point scale, in which moderate and severe impairment were combined; ‘Hygiene’ was rescored on a 3-point scale combining mild/moderate/severe; ‘Doing hobbies’ was scored on a 4-point scale, with mild/moderate impairment combined and severe retained as a separate category. MDS-UPDRS assessments performed from screening to month 24 in the PPMI early-PD cohort, excluding any performed after initiation of ST, were used for this analysis (the same assessments as those used in the primary analysis; *N* = 1906). The ‘anchored’ approach used in this analysis allowed estimation of the most likely responses of participants to the items assessing the impact on their daily lives, depending on the overall severity of the targeted motor signs.

## Supplementary information

Supplementary material

## Data Availability

This manuscript is a secondary analysis of the data from the publicly available PPMI observational study. Data from PPMI can be accessed by submitting an online application to PPMI.
