# Supplementary material for: Optimizing the MDS-UPDRS Part III for early-stage Parkinson’s: early supportive evidence for a limb-related bradykinesia/rigidity sub-score
Source: NPJ Parkinsons Dis. 2025 Oct 17;11:297. doi: 10.1038/s41531-025-01072-2 (PMC12534395; doi:10.1038/s41531-025-01072-2)
Supplement: Supplementary file 1 — Supplementary material [file 41531_2025_1072_MOESM1_ESM.pdf]

Supplementary material

Optimizing the MDS-UPDRS Part III for early-stage Parkinson's: early supportive evidence for a limb-related bradykinesia/rigidity sub-score

Running title: MDS-UPDRS Part III sub-score for early-stage PD

Antoine Regnault[1], Maria Key Prato[2], Stéphane Quéré[1], Anne Benoit[2], Nathalie J. Massat[3], & Thomas Morel[4,*]

[1]Department of Statistics, Modus Outcomes, A THREAD company, Lyon, France.

[2]Department of Biometrics and Data Sciences (BDS), UCB, Brussels, Belgium.

[3]Department of Statistics, UCB (contracted via Veramed Ltd, Twickenham), Slough, United Kingdom.

[4]Department of Patient Centred Outcomes Research, UCB, Bulle, Switzerland.

*Correspondence to: Thomas Morel, UCB, Chemin de Croix-Blanche 10, 1630 Bulle, Switzerland. E-mail: thomas.morel@ucb.com

Supplementary Table 1 | Individual item fit of the MDS-UPDRS Part III 'rest tremor' item set

Item	Fit residual	Chi-squared	*p* value
Upper-limb ipsilateral rest tremor amplitude	–7.763	15.944	0.0256
Upper-limb contralateral rest tremor amplitude	1.139	49.809	**< 0.0001**
Lower-limb ipsilateral rest tremor amplitude	1.212	69.070	**< 0.0001**
Lower-limb contralateral rest tremor amplitude	–1.096	16.313	0.0224
Lip/jaw rest tremor amplitude	0.820	38.976	**< 0.0001**
Constancy of rest tremor	–11.109	169.082	**< 0.0001**

Fit residuals recommended range is –2.5 to +2.5; values highlighted in green are <–2.5 indicate items over-discriminating the measured concept.

Chi-square probabilities were reported before Bonferroni adjustment; those significant after Bonferroni adjustment at $p < 0.01$ are shown in bold.

N = 1,906 MDS-UPDRS assessments in 423 patients.

MDS-UPDRS Movement Disorder Society Unified-Parkinson's Disease Rating Scale.

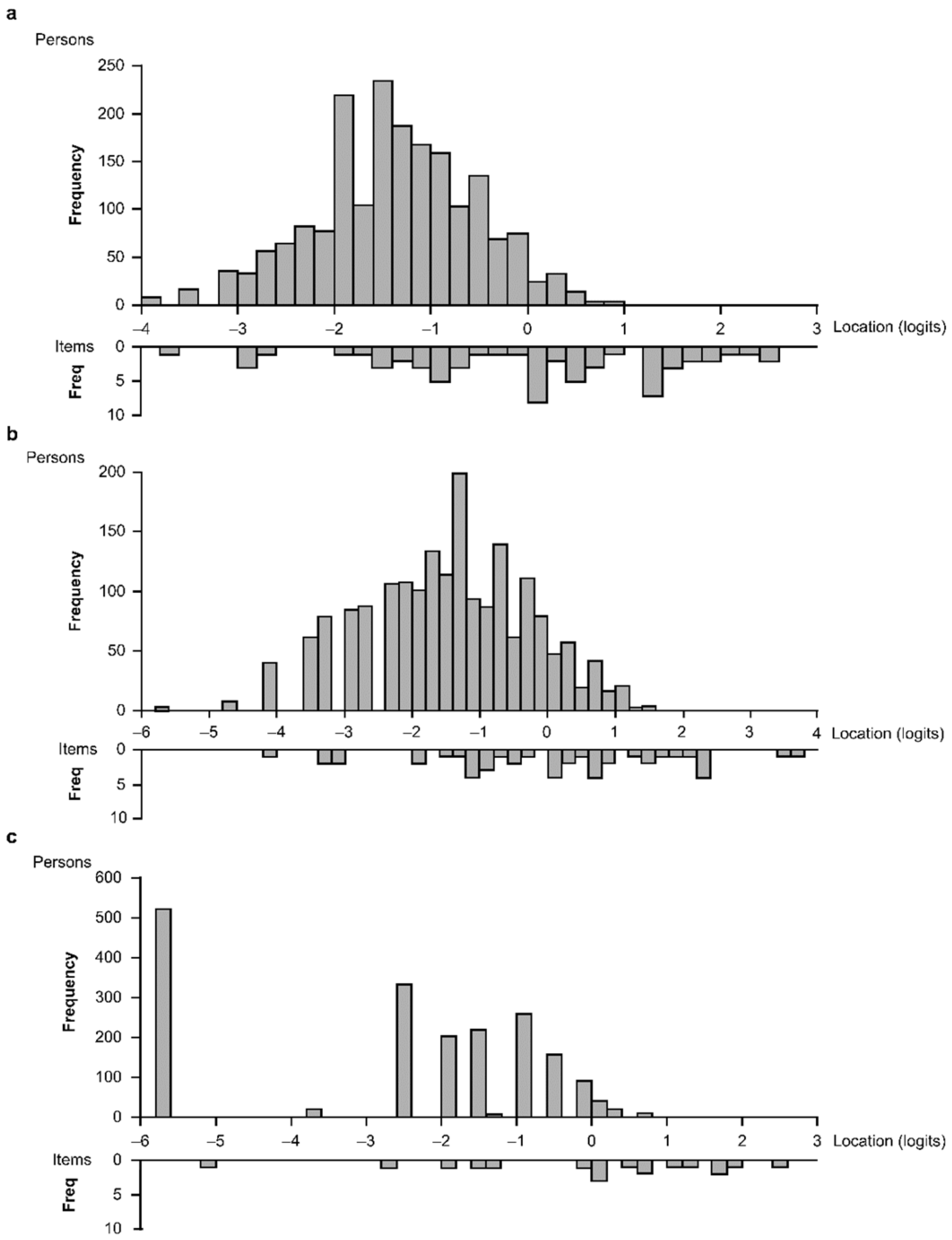

Supplementary Fig. 1 | Scale-to-sample targeting of the MDS-UPDRS Part III item sets relating to bradykinesia, rigidity, and rest tremor (21 items; a); bradykinesia and rigidity (15 items; b); and rest tremor (6 items; c).

Based on MDS-UPDRS assessments in participants with untreated (ST-naïve) PD (pooled data from assessments performed at screening, baseline, month 3, month 6, month 9, month 12, month 18, and month 24; *N* = 1,906). The upper panels show the distribution of the individual observations over the continuum of symptom severity; the lower

panels show the distribution of the item thresholds, i.e., the boundaries between adjacent item response categories, on the continuum of symptom severity. The best match is achieved with the 15-item bradykinesia and rigidity item set (**b**) although some gaps in the coverage of the milder motor signs persists.

Freq frequency, *MDS-UPDRS* Movement Disorder Society-Unified Parkinson's Disease Rating Scale, *PD* Parkinson's disease, *ST* symptomatic treatment.

Plain language summary

Being able to accurately measure how Parkinson's progresses is important when testing potential treatments for people with Parkinson's. This is especially important for disease-modifying therapies that are tested in people with early-stage Parkinson's.

The Movement Disorder Society Unified-Parkinson's Disease Rating Scale (MDS-UPDRS) measures changes in Parkinson's symptoms and progression. This scale is used in clinical trials; however, the MDS-UPDRS was not specifically made for early-stage Parkinson's. Many of the symptoms tested (also called items) of the MDS-UPDRS are not relevant to people with early-stage Parkinson's.

Our aim was to develop and test a sub-score of MDS-UPDRS containing items that reflect symptoms important to people with early-stage Parkinson's. This sub-score should also be able to measure Parkinson's progression more accurately in the early stages than the total MDS-UPDRS score.

We looked at data from 423 participants with untreated, early-stage Parkinson's in the Parkinson's Progression Markers Initiative (PPMI). We assessed 21 MDS-UPDRS items previously shown to be important to people with early-stage Parkinson's. These items were related to slow movement ('bradykinesia'), stiff, inflexible muscles ('rigidity') of the limbs, and involuntary shaking of parts of the body when resting ('resting tremors'). We found that 15 of

these items related to slow movement and stiff, inflexible muscles of the limbs worked well to measure disease progression in early-stage Parkinson’s; resting tremor items did not.

A sub-score of these 15 MDS-UPDRS items could possibly be used in future clinical trials of early-stage Parkinson’s, to assess if treatments can slow down the progression of Parkinson’s.
